# Prevalence of monoclonal gammopathy of undetermined significance (MGUS) at HIV diagnosis in individuals 18–40 years old: a possible HIV indicator condition

**DOI:** 10.1038/s41408-021-00489-1

**Published:** 2021-05-16

**Authors:** Michele Bibas, Silvia Pittalis, Nicoletta Orchi, Gabriella De Carli, Chiara Agrati, Enrico Girardi, Andrea Antinori, Vincenzo Puro, Giuseppe Ippolito

**Affiliations:** National Institute for Infectious Diseases “Lazzaro Spallanzani”, Rome, Italy

**Keywords:** Haematological diseases, Immunological disorders

Dear Editor,

Near 25% of the 38 million of people living with human immunodeficiency virus (HIV) infection are unaware of their HIV status^1^. Late diagnosis reduces treatment outcomes and increases healthcare costs and transmission rates. Routine testing for conditions that are considered indicators in an HIV prevalence of >0.1% is recognized to be cost-effective and has the potential to increase earlier diagnosis of HIV^2^.

Among the fourteen indicators condition (IC) identified for an effective strategy to diagnose people living with HIV, the only laboratory hematological parameters included are the unexplained leukocytopenia or thrombocytopenia of at least 4-weeks duration^3^.

In clinical practice, we frequently observe hypergammaglobulinemia with monoclonal or oligoclonal proteins in the serum of newly diagnosed HIV patients.

As well know, the monoclonal gammopathy of undetermined significance (MGUS) is found in more than 3.2% and 5.3% of the general population aged more than 50 and 70 years, respectively^4^. In contrast, the prevalence reported in individuals younger than 40 years is 0.3%, representing a very small subset of all MGUS^5^.

A variety of risk factors have been associated with the development of MGUS most of them are nonmodifiable and include age, male sex, black race, pesticide exposure, and family history of MGUS and related disorders. Furthermore, the risk of developing MGUS may be also associated with prior bacterial or viral infections especially in immunocompromised patients^6^.

A higher prevalence rate of MGUS has been reported in HIV-infected persons, ranging between 3 and 26%. Compared to the general population, MGUS in HIV-infected patients occurs at much younger age and, often disappers after effective antiretroviral treatment (ART)^7–9^.

There is little evidence regarding the prevalence of MGUS in young adults especially at HIV diagnosis time. We therefore wondered, eliminating the age bias, whether the presence of a monoclonal gammopathy could be helpful in indicating a possible HIV infection.

Therefore, the first aim of this study was to determine the prevalence rate of serum MGUS at HIV infection diagnosis in individuals aged 18–40. We further compared, in the same cohort at HIV diagnosis, the prevalence of MGUS with the prevalence of thrombocytopenia (TP), a consolidated indicator condition of undiagnosed HIV.

We performed a retrospective cohort study involving 1787 nonselected adult patients (18–40 years old) with newly diagnosed HIV infection admitted at the National Institute for Infectious Diseases “Lazzaro Spallanzani” from January 2009 through December 2019.

From the original study population of 1787, 39 patients were excluded from the analysis: 15 for previous or concomitant hematologic malignancies, 14 for previous TP, and 10 for previous MG. Then, 1748 individuals, 1444 (82.6%) males and 304 (17.4%) females were assessed.

Serum protein immunofixation, to confirm MGUS, in suspected agarose-gel-electrophoresis, was performed on all subjects within 1 month from confirmed HIV-positive test. In the same blood samples, TP, defined as platelet count below <100,000/mm^3^ lasting for 4 weeks, was investigated by routinary blood cell count test and confirmed in at least two consecutive determinations.

As result, MGUS was found in 77 patients, 60 males (77.9%) and 17 females (22.1%) with an overall prevalence rate of 4.40% (95% confidence interval (CI), 3.5–5.5). (Table 1). Regarding race distribution, 62 patients were white, 13 were black, and 2 were asian. The median concentration of serum M protein was 0.8 g/L (range <3 g/L). The isotype of the monoclonal immunoglobulin was IgG in 58 (75.3%), IgM in 12 (15.7%), and IgA in 7 (9%). The serum light-chain type was kappa in 43 (55.8%) and lambda in 34 (44.2%). The concentration of uninvolved immunoglobulins was normal in all patients. Neither biclonal M protein nor light chain were found. Urine from subjects with MGUS was not tested.

**Table 1:** Patients’ characteristics^a^.

Variable	MG	TP	*P*-value
Total, no. (%) (95% CI)	77 (4.40%) 3.5–5.5	20 (1.14%) 0, 7–1.8	0.001
Median age, (range)	32 (21–40)	34.5 (24–40)	
Male sex, no. (%)	60 (77.9%)	16 (80%)	
Late presenters, no. (%) (95% CI)	42 (54.5%) 1.7–3.2	18 (90%) 0.7–1.8	
HCV coinfection, no. (%)	5 (6.4%)	4 (20%)	
MG concentration, median	<0.8 g/L		
Severe TP (PTLS < 25.000/mm^3^), no. (%)		2 (10%)	
Immunoglobulin isotype, no. (%)			
IgG	58 (75.3%)		
IgM	12 (15.7%)		
IgA	7 (9%)		
Light-chain type, no. (%)			
Kappa	43 (55.8%)		
Lambda	34 (44.2%)		

Late presenters: defined as patients with a CD4 count < 350 cells/mm^3^ or AIDS at HIV diagnosis. Severe TP: defined as platelet count below <25.000/mm^3^.

*C.I.* confidence interval, *MG* monoclonal gammopathy, *TP* thrombocytopenia, *PLTS* platelets, *HCV* Hepatitis C virus.

^a^1748 individuals, 1444 (82,6%) males and 304 (17.4%) females were assessed. The study population consisted primarily of 1787 newly diagnosed HIV adults of 18–40 years old. Of them 39 patients were excluded: 15 for previous or concomitant hematologic malignancies, 14 for previous TP, 10 for previous MG.

TP was found in 20 patients, 16 males (1.11%) and 4 females (1.31%), with an overall prevalence rate of 1.14%, (95% C.I. 0.7–1.8%). Severe TP (defined as platelet count below 25,000/mm^3^) was found in two patients (10%) whereas the median value of TP was 60,000/mm^3^. Of note, none of the MGUS patients showed thrombocytopenia.

Prevalence rate of late presentation (defined as newly HIV patients with a CD4 count < 350 cells/mm^3^) was not higher in the MGUS group: 42 patients (54.5%) [2.40% (95% C.I. 1.7–3.2)] over 77 [(4.40% (95% C.I. 3.5–5.5)]. In contrast, late presenters were near the majority in the thrombocytopenic group: 18 (90%) [1% (0.6–1.6) over 20 [1.14% (95% C.I. 0.7–1.8)]. Regarding hepatitis C virus (HCV) in this population, coinfection was found in 5/77 (6.4%) of MGUS group and in 4/20 (20%) of TP patients.

Our study explored the MGUS prevalence rate at time of HIV diagnosis in 1787 patients younger than 40 years of age. We showed that prevalence of MGUS is remarkably higher in newly diagnosed young HIV patients compared with the general population. Further, in those patients, MGUS prevalence rate is significantly more common than TP prevalence, one among the consolidated indicator condition of undiagnosed HIV infection. In summary, we strongly suggest that any person younger than 40 with a MGUS should be recommended HIV testing and we think that including MGUS as indicator alteration could be useful to reduce late diagnosis of HIV in people younger than 40 years old.
