# Publisher Correction: Prevalence of monoclonal gammopathy of undetermined significance (MGUS) at HIV diagnosis in individuals 18–40 years old: a possible HIV indicator condition

**DOI:** 10.1038/s41408-021-00501-8

**Published:** 2021-06-02

**Authors:** Michele Bibas, Silvia Pittalis, Nicoletta Orchi, Gabriella De Carli, Chiara Agrati, Enrico Girardi, Andrea Antinori, Vincenzo Puro, Giuseppe Ippolito

**Affiliations:** National Institute for Infectious Diseases “Lazzaro Spallanzani”, Rome, Italy

**Keywords:** Haematological diseases, Immunological disorders

Correction to: *Blood Cancer Journal*

10.1038/s41408-021-00489-1 published online 16 May 2021

In the original article, an error occurred in the names, all family names are in place of the first names, even if the Orcid connection is right.

The correct author names are:

Michele Bibas, Silvia Pittalis, Nicoletta Orchi, Gabriella De Carli, Chiara Agrati, Enrico Girardi, Andrea Antinori, Vincenzo Puro & Giuseppe Ippolito

The original article has been updated.

